# Historical overview and update on relapsing fever group *Borrelia* in Latin America

**DOI:** 10.1186/s13071-022-05289-5

**Published:** 2022-06-08

**Authors:** Álvaro A. Faccini-Martínez, Carlos Ramiro Silva-Ramos, Adriana M. Santodomingo, Alejandro Ramírez-Hernández, Francisco B. Costa, Marcelo B. Labruna, Sebastián Muñoz-Leal

**Affiliations:** 1 Research Institute, Fundación Universitaria de Ciencias de la Salud - FUCS, Bogotá, Colombia; 2 Servicios y Asesorías en Infectología - SAI, Bogotá, Colombia; 3 Latin American Group for the Study of Ornithodoros-borne Borrelioses (Grupo Latinoamericano Para el Estudio de Borreliosis Transmitidas Por Ornithodoros [GLEBTO]), Bogotá, Colombia; 4 Grupo de Enfermedades Infecciosas, Departamento de Microbiología, Facultad de Ciencias, Pontificia Universidad Javeriana, Bogotá, Colombia; 5 Department of Animal Science, Faculty of Veterinary Sciences, University of Concepción, Chillán, Ñuble Chile; 6 Grupo Parasitología Veterinaria, Departamento de Salud Animal, Facultad de Medicina Veterinaria y de Zootecnia, Universidad Nacional de Colombia, Bogotá, Colombia; 7 Faculdade de Medicina Veterinária, Universidade Estadual Do Maranhão, São Luís, MA Brazil; 8 Departamento de Medicina Veterinária Preventiva e Saúde Animal, Faculdade de Medicina Veterinária e Zootecnia, Universidade de São Paulo, São Paulo, Brazil

**Keywords:** Soft ticks, *Ornithodoros*, Spirochetes, Tick-borne diseases, Relapsing fever, *Borrelia*, Clothing lice, Latin America

## Abstract

Relapsing fever group *Borrelia* (RFGB) are motile spirochetes transmitted to mammalian or avian hosts through the bite of hematophagous arthropods, such as soft ticks (Argasidae), hard ticks (Ixodidae) and the human clothing lice. RFGB can infect pets such as dogs and cats, as well as birds, cattle and humans. *Borrelia recurrentis*, *B. anserina* and *B. theileri* are considered to have worldwide distribution, affecting humans, domestic birds and ruminants, respectively. *Borrelia* spp. associated with soft ticks are transmitted mainly by *Ornithodoros* ticks and thrive in endemic foci in tropical and subtropical latitudes. Nowadays, human cases of soft tick-borne relapsing fever remain neglected diseases in several countries, and the impact these spirochetes have on the health of wild and domestic animals is largely understudied. Human infection with RFGB is difficult to diagnose, given the lack of distinguishing clinical features (undifferentiated febrile illness). Clinically, soft tick or louse-borne relapsing fever is often confused with other etiologies, such as malaria, typhoid or dengue. In Latin America, during the first half of the twentieth century historical documents elaborated by enlightened physicians were seminal, and resulted in the identification of RFGB and their associated vectors in countries such as Mexico, Panama, Colombia, Venezuela, Peru and Argentina. Almost 80 years later, research on relapsing fever spirochetes is emerging once again in Latin America, with molecular characterizations and isolations of novel RFGB members in Panama, Bolivia, Brazil and Chile. In this review we summarize historical aspects of RFGB in Latin America and provide an update on the current scenario regarding these pathogens in the region. To accomplish this, we conducted an exhaustive search of all the published literature for the region, including old medical theses deposited in libraries of medical academies. RFGB were once common pathogens in Latin America, and although unnoticed for many years, they are currently the focus of interest among the scientific community. A One Health perspective should be adopted to tackle the diseases caused by RFGB, since these spirochetes have never disappeared and the maladies they cause may be confused with etiologies with similar symptoms that prevail in the region.

**Graphical Abstract:**

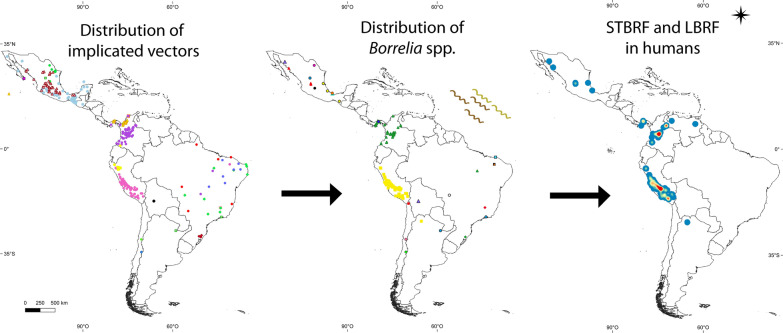

**Supplementary Information:**

The online version contains supplementary material available at 10.1186/s13071-022-05289-5.

## Background

Spirochetes in the family *Borreliaceae* are host-associated agents that infect ticks, louse and vertebrates [1, 2]. Recently, a still controversial proposition splits the family into two genera: *Borrelia*, consisting of those species that cause relapsing fever, and *Borreliella*, covering Lyme borreliosis (*Borrelia burgdorferi* sensu lato [s.l.]) species [1, 2]. Pathogenic relapsing fever group *Borrelia* (RFGB) comprise motile bacteria, 0.2–0.5 µm in diameter and 10–40 µm in length, that thrive in transmission cycles involving soft ticks (*Ornithodoros*, *Argas*), hard ticks (*Rhipicephalus*, *Ixodes*) and the human clothing lice *Pediculus humanus humanus* as their vectors [1, 2]. RFGB replicate profusely in the blood of competent hosts and achieve transovarial transmission in ticks [3]. Some RFGB cause illness in pet animals such dogs and cats, as well as in birds, cattle and humans [1–4]. Twenty-one species of RFGB are currently considered recognized, occurring in both temperate and tropical countries. *Borrelia recurrentis* (transmitted by *P. humanus humanus*), *Borrelia anserina* (transmitted by *Argas* spp.) and *Borrelia theileri* (transmitted by *Rhipicephalus* spp.) can be considered to be distributed worldwide, and infect humans, birds and domestic ruminants, respectively [4, 5]. Conversely, human pathogenic RFGB transmitted by *Ornithodoros* ticks exist across specific geographical areas that are defined by the distribution of the vector tick [5]. The main species present in Africa are *Borrelia crocidurae* and *Borrelia duttonii* (transmitted by *Ornithodoros sonrai* and *Ornithodoros moubata*, respectively); in the Mediterranean region, *Borrelia hispanica* (transmitted by ticks of the *Ornithodoros erraticus* complex); in Asia and Eurasia, *Borrelia latyschewii* and *Borrelia persica* (transmitted by *Ornithodoros tartakovskyi* and *Ornithodoros tholozani*, respectively); and in North America, *Borrelia hermsii*, *Borrelia turicatae* and *Borrelia parkeri* (transmitted by *Ornithodoros hermsi*, *Ornithodoros turicata* and *Ornithodoros parkeri*, respectively) [5, 6]. Importantly, RFGB include *Borrelia miyamotoi*, a unique species transmitted by ticks of the *Ixodes ricinus* complex that is of medical importance in temperate regions of the Northern Hemisphere [7].

Nowadays, the diagnosis of RFGB infection is still deficient with many neglected cases, so the impact that the spirochetes have on animals or humans is largely unknown [4, 5]. In terms of human health, soft tick- and louse-borne relapsing fever spirochetes remain largely unsuspected as etiological agents in Latin America because clinical symptoms mirror more common maladies, such as malaria, typhoid and dengue, hampering the diagnosis in regions that lack specific laboratory assays [8].

In Latin America, historical data published during the first half of the twentieth century supported the occurrence of RFGB infection in humans. At this time, seminal work described novel RFGB species (*Borrelia mazzottii*, *Borrelia dugesii*, *Borrelia venezuelensis*), documented species for the first time in the region (*B. recurrentis*, *B. turicatae*) and identified local vectors (*O. turicata*, *Ornithodoros talaje*, *Ornithodoros dugesi*, *Ornithodoros rudis* and *P. humanus humanus*) in Mexico, Panama, Colombia, Venezuela and Peru [5, 9, 10]. After a gap of 80 years, interest in RFGB has re-emerged among scientists and physicians.

This review constitutes an overview of RFGB in Latin America, and includes data retrieved from old medical manuscripts that bring to light forgotten epidemiological aspects of the diseases in the continent. We present a narrative on RFGB for different countries where information regarding the disease was available. Given that our discussions include medical terminology, we first describe the clinical and epidemiological features of soft tick- and louse-borne relapsing fevers, as well as laboratory diagnosis in humans. Data on RFGB related to animals in Latin America are summarized. We show maps constructed with the program QGIS v 3.18.1-Zürich (www.gnu.org/licenses) using centroids established with Google Earth Pro v 7.3.4.8248 for those records where the specific locations were not reported. Heat maps were constructed upon layers of georeferences of the localities where human relapsing fever cases were reported, using the QGIS Heatmap algorithm with modification of the radius parameter. The algorithm calculates the density based on the number of location points, so that the greater the number of points in a given region, the higher the density.

## Soft tick-borne relapsing fever

Soft tick-borne relapsing fever (STBRF) is a zoonotic disease with worldwide distribution, transmitted to humans by ticks in the genus *Ornithodoros* [5, 6]. Pathogenic spirochetes are maintained in enzootic cycles involving both soft ticks and small mammals as reservoirs. *Ornithodoros* ticks are fast-feeding nidicolous parasites that become infected while sucking blood with spirochetes and they remain infected for several weeks to years [5]. Humans are accidentally infected with RFGB when exposed to environments where populations of *Ornithodoros* spp. are established, such as bird nests, rocky environments, caves or human dwellings [5].

The incubation period ranges from 4 to 18 days after the bite of an infected tick [11], and patients typically develop an abrupt fever onset (38.7–40 °C) [5, 6]. The first febrile episode, which is commonly accompanied by nonspecific symptoms such as headache, arthralgia, myalgia and nausea, is usually the longest and lasts for an average of 3 days, terminating with a crisis of shaking chills or rigors [3, 11]. A series of relapses (ranging from 1 to 13) follows the initial symptoms, each relapse corresponding to peaks of spirochetemia [5, 6]. The average time between febrile episodes is 7 days [11]. This typical pattern of recurrent fever is usually described during the course of non-fatal infections in the absence of antibiotic treatment [3, 6, 11]. Thus, STBRF should be suspected in any patient with undifferentiated febrile illness, especially if episodes of fever culminate in a crisis and if patients were exposed to *Ornithodoros* ticks [3].

Uncommon manifestations of STBRF include iritis, acute respiratory distress syndrome, uveitis, iridocyclitis, cranial nerve palsy, myocarditis and spleen rupture [11]. The severity of neurological symptoms is variable, with infections caused by *B. duttonii* and *B. turicatae* being the most neurotropic [5]. The fatality rates for untreated cases of *B. duttonii* infection ranges from 4 to 10%; however, if appropriate antibiotics are promptly supplied, the death rate is < 2% [3]. Infants and pregnant women are prone to develop severe disease [3], and infection during pregnancy frequently leads to abortion or stillbirth [12, 13].

## Louse-borne relapsing fever

Louse-borne relapsing fever (LBRF) is caused by *B. recurrentis* and restricted to one vector, the human clothing louse *P. humanus humanus*, which feeds only on humans [3, 14]. There is no evidence that mammals other than humans maintain the infection in nature [3, 14]. Lice become infected with *B. recurrentis* through a blood meal taken on a spirochetemic patient. Unlike *Ornithodoros* ticks, *B. recurrentis*-infected lice cannot infect their progeny and, therefore, they are not reservoirs of borreliae [3, 14].

Humans do not acquire LBRF through the bite or the saliva of the louse. Conversely, coelomic fluid from a crushed louse or louse feces infected with *B. recurrentis* penetrate through damaged skin or conjunctiva while scratching [3, 14]. Crowding and poor personal hygiene (e.g. refugee centers, homeless people) increase the risk of infestation by clothing lice and therefore the transmission of *B. recurrentis* [14].

As in STBRF, the incubation period of LBRF is between 4 and 18 days after the contact with an infected louse [14]. Patients develop fever that can approach 40 °C accompanied by rigors, headache, dizziness, generalized aches and pains, prostration and confusion. Meningism, hepatic enlargement, jaundice, subconjunctival hemorrhages, epistaxis and a petechial or ecchymotic rash involving the trunk are common signs [14]. In the absence of antibiotic treatment, the fever lasts for an average of 5 days, terminating with a crisis; a series of relapses (range: 1–5) follows with afebrile remissions of 5–9 days between each episode [3, 14]. LBRF must be distinguished from other louse-borne bacterial infections that trigger an undifferentiated febrile illness, such as trench fever and epidemic typhus, caused by *Bartonella quintana* and *Rickettsia prowazekii*, respectively [3, 14].

Severe manifestations may include coma, shock, myocarditis, acute respiratory distress syndrome, hepatic failure, spleen rupture and disseminated intravascular coagulation leading to intracranial, gastrointestinal or pulmonary hemorrhages [14]. Fatality rates for untreated disease range from 10 to 70%; yet a prompt treatment with appropriate antibiotics diminishes the death rate to 2–5% [3]. As in STBRF, pregnant women are especially susceptible to developing severe disease, with abortion or stillbirth a frequent result [14]. A comprehensive review on LBRF was recently published, and it is recommended for further epidemiological details [15, 16].

## Laboratory diagnosis

Blood anomalies in patients with STBRF and LBRF are unspecific. Moderate normochromic, normocytic anemia, neutrophil leukocytosis and thrombocytopenia are common signs [3, 14]. The erythrocyte sedimentation rate and serum concentrations of aminotransferases are often moderately elevated [3]. Analyses of the cerebrospinal fluid (CSF) usually indicate meningeal inflammation with mononuclear pleocytosis, mildly to moderately elevated protein levels and normal glucose levels [3].

During symptomatic febrile disease, estimates of RFGB in the blood range from 10^5^ to 10^6^ spirochetes/ml; in contrast, infections with the *B. burgdorferi* s.l. group does not surpass 10^4^ spirochetes/ml [11, 17]. Thus, the gold standard diagnosis for relapsing fever is direct microscopic visualization of borreliae, as a density of at least 10^4^–10^5^ spirochetes per milliliter of blood is easily seen [11, 18]. Thick and thin blood smears should be taken while patients are febrile, stained with Giemsa, Wright, or Diff-Quick or examined under dark-field microscopy [3, 14]. Once the temperature of an untreated patient declines, spirochetes vanish and their visualization is often impossible [3, 14]. A two-stage centrifugation step to concentrate the sample may help to visualize spirochetes when present at < 10 spirochetes per milliliter blood [19]. Thus, the factors that may hinder the detection of spirochetes in peripheral blood smears are: (i) the microscopist’s experience; (ii) the lack of suspicion of the disease; (iii) the increased use of automated instruments for blood cell counts; and (iv) the examination of blood in the asymptomatic interval [11].

RFGB are fastidious slow-growing spirochetes, and isolation attempts require specialized liquid media, such as Barbour-Stoenner-Kelly (BSK-II) or Modified Kelly-Pettenkofer (MKP) supplemented with high serum concentrations [6, 7, 14, 20]. A novel formulation of the BSK broth (BSK-R) has been developed recently [21], and its application with uncultured RFGB is promising. With this new formulation, a few drops of clinical samples (e.g. patient’s blood or plasma) obtained during the febrile period are inoculated into the broth, incubated at 34–35 °C and then examined for spirochetes by dark-field microscopy 2–6 weeks post-inoculation [3, 11]. Animal inoculation or xenodiagnosis (to feed presumed infected ticks upon laboratory animals) has been used to recover the spirochetes before cultivation in axenic media [20]. The inoculation of blood, plasma, or CSF into laboratory rodents may amplify the number of spirochetes to a detectable level in the animal’s blood, even when the blood sample was obtained from a patient during an afebrile period [3, 11]. In this case, the blood of the inoculated animal should be examined daily for the presence of spirochetes for at least 10 days post-inoculation [3].

Serological confirmation is demonstrated with a fourfold rise of antibody titer between the acute and convalescent phases of infection, as determined by enzyme-linked immunoassay (ELISA) or indirect immunofluorescence assay (IFA) [11]. Patients previously infected with *B. burgdorferi* s.l. may yield false positive reactions when whole-cell lysates of cultured bacteria are used, mainly because of the similarity of epitopes on the spirochetes’ flagellin protein [11]. GlpQ (glycerophosphodiester phosphodiesterase)- and BipA (*Borrelia* immunogenic protein A)-specific antigens, which are shared by all RFGB but absent in the *B. burgdorferi* s.l. group, are recommended to avoid cross reactions between different groups of *Borrelia* spp. [6, 20].

Molecular diagnosis by PCR and sequencing of amplicons offer a number of advantages to detect and identify species-specific *Borrelia* infections in cases where the microorganism is difficult to cultivate [3]. PCR is more sensitive than microscopy, and the results can be obtained within few hours [3, 20]. Conserved genes, such as* 16S* ribosomal RNA (rRNA), *flaB* (flagellin) and *glpQ* are usually targeted for diagnosis [6, 18, 20]. The major limitation of this approach is obtaining sufficient borrelial DNA from a given sample for the analysis. Extracting DNA from blood and/or CSF collected during the febrile episodes generally yields positive results [20].

## RFGB in domestic animals

*Borrelia anserina*, *B. theileri* and other *Borrelia* species have been described as agents of disease in birds, cattle and other domestic animals such as dogs and cats [4]. In dogs, STBRF caused by *B. turicatae* and *B. hermsii* has been reported in the USA [22–24]. Cats and dogs infected by *B. persica* were reported in Iran and Israel [25, 26], and with *B. hispanica* in Spain [27]. Overall, STBRF produces lethargy, anorexia, anemia and thrombocytopenia in all infected animals, while fever seems to be more frequent in dogs than in cats [25–27]. Interestingly, a recent study used dogs without exposure to *B. burgdorferi* but experimentally infected with *B. turicatae*, and showed that generated antibodies cross-reacted with serological assays (whole-cell IFA test and multi-antigen tests) designed to detect *B. burgdorferi*, the causative agent of Lyme borreliosis [28]. These results suggest that a critical evaluation is needed when performing diagnostic tests aiming at the identication of *B. burgdorferi* exposure in dogs coming from outside Lyme borreliosis endemic areas, and that RFGB should be considered as possible etiological agents in positive tests [28].

*Borrelia anserina* is transmitted mainly by the soft ticks *Argas persicus* and *Argas miniatus*, and is the causative agent of avian borreliosis [4, 29, 30], a highly fatal septicemic disease of hens, geese, ducks and turkeys in tropical and sub-tropical regions [4, 30]. *Borrelia anserina* may be found in the blood of infected birds during the initial stages of the disease and causes hyperthermia, polydipsia, drowsiness, anorexia, inappetence, greenish diarrhea, paralysis of the legs and wings, as well as sudden death [29].

*Borrelia theileri* is transmitted by hard ticks of *Rhipicephalus* (*Boophilus*) subgenus and is the etiological agent of bovine borreliosis [4]. The infection has also been reported in horses and sheep [4]. *Borrelia theileri* has been identified in Africa, Australia, Europe and South America [31–34]. Clinically, bovine borreliosis is a mild febrile disease associated with lethargy, hemoglobinuria and anemia [31]. Simultaneous infection with *B. theileri* and *Babesia* is common, especially on cattle introduced from areas free of *Rhipicephalus* (*Boophilus*) spp. [35]. Detection of *B. theileri* in thin blood films is not common due to the low spirochetemia that this borrelia develops [36]. Serological cross-reactivity between genospecies belonging to the *B. burgdorferi* s.l. group and *B. theileri* has been described [37], thus studies of *B. burgdorferi* seroprevalence in cattle from areas non-endemic for Lyme borreliosis should be carefully interpreted [34].

## Historical overview of RFGB in Latin America

### Relapsing fever in Mexico

Soft tick-borne relapsing fever was once suspected to occur in Mexico given the wide geographical distribution of two vectors, *O. talaje* and *O. turicata*, but it remained undiagnosed for a long time. It was not until 1936 that Pilz and Mooser confirmed the disease in Aguascalientes city (Aguascalientes State) after examining a thick blood smear that revealed spirochetes in a patient with malaria-like symptoms: four fever relapses with severe headaches, myalgias, chills, mild jaundice, hepatomegaly and leukocytosis with neutrophilia [38]. Subsequently, the same authors diagnosed novel cases at the same locality in two persons living in houses next to a barnyard infested by rodents and *O. turicata* ticks. At this time, the authors were able to perform the first isolation of a Mexican RFGB using blood samples of one patient and also by inoculating macerated soft ticks into white rats [38]. One year later, Martínez Rivas reported 18 additional cases in the same city [39].

Intrigued by these discoveries, the Mexican physician Luis Mazzotti and co-researchers conducted extensive field work across several Mexican states between 1938 and 1953, collecting *Ornithodoros* species with the aim to demonstrate their role as vectors of RFGB. Using laboratory animals, they were able to recover spirochetes from at least three *Ornithodoros* species: *O. turicata*, collected in the states of Puebla, México, Aguascalientes, Guanajuato, San Luis Potosí, Michoacán, Querétaro, Coahuila de Zaragoza, Sinaloa and Jalisco [40–42]; *O. talaje*, collected in the states of Guerrero, Chiapas, Veracruz and Oaxaca [41]; and *O. dugesi* collected in Coahuila de Zaragoza State [43]. At the time, three species of *Borrelia* were described in association with each of the three soft tick species, and are currently known as *B. turicatae*, *B. mazzottii* and *B. dugesi* [40, 44–46].

In 1944 and 1946, other confirmed human cases were described in Jalisco and Veracruz States, respectively [47, 48]. The patient in Jalisco was bitten by numerous ticks while staying overnight inside a horse barn in the city of Encarnación Díaz. One week later he developed an initial febrile episode lasting for 5 days, accompanied by malaise, intense headache, myalgias and chills. He presented three relapsing episodes and the treatment against malaria failed. Blood samples collected during the last febrile episode were inoculated into white rats, and spirochetes were recovered, as observed in Giemsa-stained thin smears 6 days post-inoculation. The patient was cured after treatment with neosalvarsan. Days later, the patient provided researchers with ticks from the horse barn, which were identified as *O. turicata*; the infection with RFGB was subsequently confirmed using laboratory animals [47]. In Veracruz, three cases of suspected malaria were confirmed to be spirochetosis after the visualization of bacteria in blood smears. The infections were acquired at locations in Apazapan and Paso Real municipalities. The three patients were satisfactorily treated with penicillin [48].

Davis published a paper in 1956 in which he proposed *B. mazzottii* as the RFGB species related to *O. talaje* [46], but research on STBRF in Mexico then vanished for more than 50 years. However, in the last decade, two probable human cases were described in Sonora State in 2012 and 2019, respectively. The former was a 12-year-old girl from Hermosillo city who presented relapsing fever during 3 months, with febrile episodes lasting 3–5 days, headache, rash, photophobia, chills, diaphoresis, weakness, epistaxis and cervical adenitis and 15- to 21-day-intervals of apyrexia. Two days before the onset of the disease she spent 6 days in a cabin. Results of laboratory tests were normal or negative, with no detection of *B. burgdorferi* antibodies; nevertheless, peripheral blood smears stained with Wright and Warthin-Starry methods showed spirochetes. Erythromycin was used as the first treatment, but this was suspended to continue with penicillin; however, the patient developed headache, fever, profuse sweating, myalgia, arthralgia, weakness and nasal obstruction. She was then treated with penicillin, ceftriaxone and doxycycline but developed a Jarisch-Herxheimer reaction; consequently, acetaminophen and glucose were administered [49]. In 2019, a 45-year-old woman with a history of outdoor activities and contact with animals in the rural area of Etchojoa Municipality exhibited five febrile episodes associated with arthralgias, diaphoresis, asthenia, adynamia, fatigue, headache, eye pain, tachycardia, abdominal pain, dyspnea, generalized diffuse rash, insomnia, nocturnal diaphoresis, daytime drowsiness and character changes. Serological tests for leptospirosis, syphilis and Lyme borreliosis were negative, and the patient received treatment with amoxicillin for 15 days without improvement. Ultimately, examination of a blood smear by dark-field microscopy unveiled circulating spirochetes. She was satisfactorily treated with doxycycline for 14 days. Suspecting STBRF, a serum sample was evaluated for reactivity against *B. turicatae* using recombinant *Borrelia* GlpQ and BipA antigens; the sample was found to be positive for both [50].

**Fig. 1:**
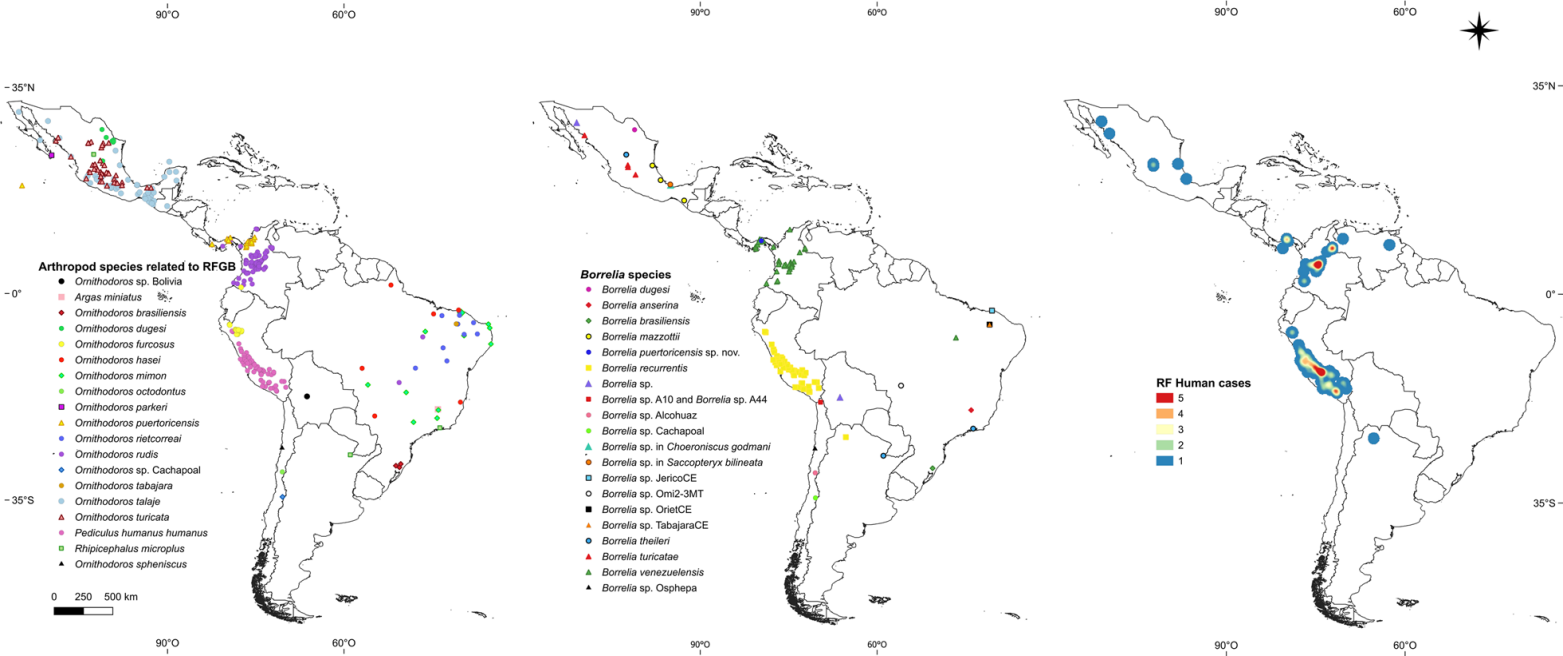
Maps of Latin America showing arthropod species implicated or putatively implicated in the transmission of RFGB, species of *Borrelia *associated with human cases, and number of human cases based on literature reports. Abbreviations: RFGB, relapsing fever group *Borrelia*; RF, relapsing fever

A recent publication by Guzmán-Cornejo et al. described the geographical distribution of soft tick species in Mexico [51]. It is likely that *Ornithodoros* spp. as vectors of RFGB are distributed as follows (Fig. 1; Additional file 1: Table S1): *Ornithodoros turicata* in the states of Puebla, Aguascalientes, Guanajuato, San Luis Potosí, Querétaro, Coahuila de Zaragoza, Jalisco, México, Durango, Guerrero, Hidalgo, Michoacán, Morelos, Nuevo León, Sinaloa, Tabasco and Zatatecas [51]; *O. talaje* in the states of Guerrero, Chiapas, Veracruz, Oaxaca, Baja California, Baja California Sur, Campeche, México, Jalisco, Michoacán, Morelos, Puebla, Quintana Roo, Sinaloa, Sonora, Tabasco, Tamaulipas and Yucatán [51]; *O. dugesi* in the states of Coahuila de Zaragoza [43], Nuevo León and San Luis Potosí [51]; *O. parkeri* in Baja California Sur State [51]; and *Ornithodoros puertoricensis* in Colima State [51].

### Relapsing fever in Panama

In 1904 and 1905, during the American occupation of the Canal Zone, it is likely that STBRF was confused with typhus or malaria [52]. Between 1905 and 1907, 31 cases were recognized in the Commission hospitals in Ancón and Colón provinces after the examination of blood smears of every patient who was admitted to the medical wards [52]. Among 17 cases occurring in 1907, more than 70% were infected in Colón province, on the Atlantic side of the isthmus [52].

A preliminary description made by Darling in 1909 considered Panamanian STBRF to be a mild disease in humans, with three to four febrile paroxysms (separated by 5–6 afebrile days), each 2–3 days, with very few spirochetes visible in blood smears [52]. Using laboratory animals, following inoculation with spirochetes, Darling described a mild infection with two spirochetemic relapses in monkeys (*Cebus* sp.) and white mice (as the most susceptible animals), and one relapse in white rats [52]. These animals were intraperitoneally inoculated with strains recovered from humans (“strain A” and “strain B”). Overall, spirochetes appeared in the animals’ blood 24 h after inoculation. Spirochetes observed in rats and mice blood measured 7.2–13.2 µm in length [52]. Interestingly, the immunity conferred by one strain was sufficient to protect the test animal against a subsequent attack with the same strain, but not enough to avoid infection with a different one [52]. Finally, attempts to infect other animals (turtle, pigeon, frog, guinea-pig, dog and goat) through inoculation of infected rat blood failed, suggesting these vertebrates as incompetent hosts [52]. A later paper written by Connor in 1917 compiled and described 17 cases diagnosed in Canal Zone hospitals from 1909 to 1917 [53].

During the last week of March and the first week of April of 1921, six American (US) males, aged between 11 and 20 years, were admitted to the Ancón Hospital with symptoms of relapsing fever [54]. Two weeks prior to admission, they had all spent several nights inside native huts in Arraiján district and were bitten by “bugs.” Larvae of *O. rudis* (misidentified as *O. talaje*) were recognized as the probable vectors [54, 55]. Suspecting STBRF, a sanitary inspection was performed in the infested dwellings, which were constructed with a mix of mud and ashes and had bamboo beds supported by black palm leaves, a thatched roof and a floor of ground [54]. The inspection team collected nearly 250 adults and nymphs and 75 unfed larvae of *O. rudis* (called “Chinche mamones” by the local inhabitants) in crevices of the black palm leaves and bamboo poles [54]. Twenty-two ticks were then macerated in saline solution and inoculated into white rats, which showed spirochetes in the blood on post-inoculation day 6 [54]. Subsequently, other experiments were carried out, demonstrating that: (1) *O. rudis* acquired the spirochetes after feeding on spirochetemic rats, and transmitted the bacteria to monkeys (*Macaccus rhesus*); (2) spirochetes were visible in tick hemolymph; (3) three human volunteers, inoculated with blood from infected white rats, a suspension of macerated ticks brought from the huts in Arraiján and bitten by ticks from the same district, respectively, became infected [54]. The three volunteers developed two febrile paroxysms and spirochetes in present in their blood; they were satisfactorily treated with arsphenamine [54]. Based on these above results, Bates and co-researchers [54] concluded that *O. rudis* was the vector of relapsing fever spirochetes in Panama.

One year later, John and Bates proved that the Panamanian spirochete was a different species entirely based on their through evaluation of the dynamics of infection in laboratory animals. These authors inoculated white rats with the Panamanian strain and other RFGB known at that time as “*Spirochete obermeieri*,” “*Spirochete novyi*,” “*Spirochete kochi*” and “*Spirochete duttoni*” [56]. Once white rats recovered from the infection, they were inoculated with heavy doses of the Panamanian strain. On post-inoculation day 8, only white rats previously infected with the Panamanian spirochete cleared spirochetes in their blood [56]. Moreover, agglutination tests demonstrated a close relationship between “*S. obermeieri*,” “*S. novyi*,” “*S. kochi*” and “*S. duttoni*,” since immune sera cross-reacted, immediately immobilizing and killing these four species, but not the Panamanian spirochete. In comparison, immune serum of the latter species caused cessation of movement, agglutination and death only of the homologous spirochetes [56]. Since the publication of this article, there has been a consensus that the “spirochete of Panama,” also named “*Spirochaeta neotropicalis*,” corresponds to *B. venezuelensis* [55, 57]. However, due to the recent isolation of a spirochete from *O. puertoricensis* (see below), the identity of Panamanian spirochetes should be carefully assessed.

Regarding preliminary hypotheses on the animal hosts implicated in the ecoepidemiology of STBRF in Panama, Darling suggested that synanthropic rats (*Rattus rattus* and *Rattus norvegicus*) could spread the spirochetes, based on the observation that they were frequently present in houses infested by *O. rudis* [58]. Thus, he assumed that rats can acquire the infection and disseminate the spirochetes from village to village [58]. However, this hypothesis was not tested at the time. Moreover, given that *O. talaje*, *O. puertoricensis* and *O. rudis* are morphologically similar, the identities of the ticks implicated in the transmission of STBRF in Panama is now obscure.

In 1930, Clark et al. conducted interesting experimental research using wild animals. Blood of squirrel-monkeys (*Leontocebus geoffroyi*) from Panama Oeste (Arraiján and La Chorrera districts) and Darién provinces, naturally infected with spirochetes, was inoculated into white mice, white rats, guinea pig, white-faced monkeys, red spider monkeys, night monkey and squirrel-monkeys [59]. The infected animals showed spirochetes that were quickly cleared from blood, with the exception of white rats, mice and squirrel-monkeys [59]. Clark et al. noted that these spirochetes (8–12 µm in length) were morphologically similar to those of species causing relapsing fever in humans [59]. This observation led to three human volunteers being experimentally infected with the squirrel-monkeys’ spirochetes through inoculation of infected blood and through the bites of *O. rudis* that had previously fed on spirochetemic monkeys. All volunteers developed relapsing fever, and the spirochetemic blood of one of them was subsequently inoculated into a juvenile squirrel-monkey that developed the disease promptly and died in about a month [59].

In 1931 and 1932, opossums (*Didelphis marsupialis*) and armadillos (*Dasypus novemcinctus*) were found to be naturally infected, with infection rates of 12% (9/77) and 6.2% (2/32), respectively [60]. Remarkably, opossums were infested with *Ornithodoros* larvae. Taking the results of Clark et al. [59] into consideration, Dunn et al. suggested that STBRF were primarily a disease of animals and that human cases most likely occurred in rural regions and were characterized by one or two paroxysms, spontaneous subsidence or even asymptomatic presence of spirochetes in blood [60].

In 1946, Calero described 106 STBRF cases in patients admitted to the Santo Tomás and Gorgas Hospitals between 1907 and 1944. Similar to Dunn et al. and Clark et al. [59, 60], he recognized that US immigrants had no immunity and that in a region with infected ticks, they could easily acquire the disease and develop typical symptoms, which were generally not observed in the native Panamanian population previously exposed to the spirochetes [61]. Regarding epidemiological characteristics of the disease in those years, Calero described a relative low incidence, with an average of 0.11%/year per 1000 hospitalizations; no specific nationality; ages of patients varying between 13 months and 50 years (82.5% between 11 and 40 years of age); and a greater incidence in males than females [61]. Clinically, he described an incubation period of 6–9 days, followed by an onset of fever, headache and violent chills lasting an average of 5 days, with 56.8% of the patients presenting one relapse (average duration: 2 days), 17.8% presenting two relapses (1 day on average), 6.3% presenting three relapses (1 day on average) and 2.1% presenting four relapses (1 day on average) (Table 1) [61]. The fever at the end of each febrile cycle always ended with a rapid crisis, accompanied by profuse perspiration. Each period of apyrexia decreased from the first to the fourth relapsing episode; 46.3% of the cases had nausea, with vomiting occurring in 41% [61]. At physical examination, the average blood pressure ranged around 110/70 mmHg, with a regular pulse full and proportional to the temperature. Splenomegaly and hepatomegaly were described in 25% and 19% of cases, respectively, and only 5% of all the patients presented icterus; 10.5% of the patients had pharyngeal congestion and three patients presented meningismus, with slight rigidity of the neck [61]. Regarding laboratory examinations, patients showed a slight anemia and leucocyte count of 8500 cells/mm^3^ on average, with a maximum and minimum leukocytosis of 16,000 and 3000 cells/mm^3^, respectively. The majority of patients were satisfactorily treated with neosalvarsan, and the prognosis was good; no deaths were reported [61].

**Table 1:** Clinical features of patients diagnosed with soft tick-borne relapsing fever in selected Latin American countries during the first half of the twentieth century

Clinical features	Panama [61]	Colombia [76]	Colombia [68]	Venezuela [90]
First author of study	Calero C	Pampana EJ	Roca García M	Pino Pou R
Number of cases	106	38	22	21
*Clinical features*				
Incubation period (days)	6–9	ND	6–8	ND
Number of febrile paroxysms (%):				
1	17	55	41	28
2	56.8	18	41	9
3	17.8	24	9	33
4	6.3	3	4	19
5	2.1	0	4	ND
Nausea (%)	46.3	ND	ND	ND
Vomiting (%)	41	70	ND	ND
Splenomegaly (%)	25	33	ND	ND
Hepatomegaly (%)	19	33	ND	ND
Icterus (%)	5	70	ND	ND
Meningismus (%)	2.8	ND	ND	ND
Headache (%)	ND	95	ND	ND
Sweating (%)	ND	91	ND	ND
Chills (%)	ND	80	ND	ND
Osteoarticular pain (%)	ND	70	ND	ND
Conjunctival injection (%)	ND	50	ND	ND

*ND* No data/no data in percentage

Following the publication Calero’s work, cases or research on STBRF became scarce in Panama. Nevertheless, in the last decade, Bermúdez et al. published records of *Ornithodoros puertoricensis* [62–66], a species described in 1947 and morphologically similar to *O. talaje*. Remarkably, collections of *O. rudis* or *O. talaje*, both vectors of spirochetes, have not been reported in Panama since early records [54, 55], yet *O. puertoricensis* seems to occur in abundance and in association with wilds animals currently. For instance, *Dasyprocta punctata* and *Eira barbara* host this tick species in Summit Municipal Park [62], and constructions housing people in two localities in Colón province (Charco La Piedra and Espinar) and one in Panama province (Ancón, Panama City) were found to be infested with this species as well [63]. Remarkably, eight persons from Charco La Piedra and one from Ancón reported symptoms compatible with toxicosis. All the collected ticks were evaluated for the presence of RFGB DNA, but no samples tested positive [63]. Contrary to these results, a novel species, *Borrelia puertoricensis*, was recently isolated from *O. puertoricensis* collected in the Summit Municipal Park, proving that this soft tick does harbor a spirochete [64]. However, any implication of *B. puertoricensis* as a human pathogen is still premature.

Considering the previously published papers, the geographical distribution of *Ornithodoros* species probably implicated as vectors of RFGB in Panama is as follows (Fig. 1; Additional file 1: Table S1): *Ornithodoros rudis* in the provinces of Panamá Oeste, Darién, Panamá and Herrera [55, 56, 58–60]; and *O. puertoricensis* in the provinces of Panamá, Colón and Chiriquí [62, 63, 65, 66].

### Relapsing fever in Colombia

The study of STBRF in Colombia represents pioneering work in South America. The disease was recognized for the first time in mid-1906 by the physician Roberto Franco, who observed spirochetes in blood smears of febrile patients living in Muzo and Villeta municipalities (Departments of Boyacá and Cundinamarca, respectively) [67, 68]. Following the publication of this finding, examining blood smears became a frequent practice among Colombian physicians treating febrile patients coming from tropical regions, and using this simple technique they were able to describe many STBRF cases [67]. One year after the cases from Muzo and Villeta occurred, STBRF was reported using this method in Manizales Municipality (Caldas Department) [69].

During late 1906 and early 1907, an undifferentiated febrile illness affected emerald mine workers in Muzo Municipality, with a fatality rate of 20% [67, 70]. Roberto Franco and co-researchers Martínez Santamaría and Toro, all physicans, were recruited at that time to study the outbreak. They first suspected malaria as a cause of the disease, yet after 6 weeks of inquiries, and based on clinical, laboratory and microscopy evidence, they concluded that the outbreak had a mixed etiology, and described 17 cases of sylvatic yellow fever and four cases of STBRF [67, 70]. Both febrile diseases were associated with chills, headache, myalgia, conjunctival hyperemia, nausea and vomiting. However, hepatosplenomegaly, repetitive chills, rapid drop of body temperature, uveitis, leukocytosis and non-fatality were more frequent in the STBRF cases [67]. Franco highlighted the usefulness of a microscope to identify spirochetes circulating in blood, which were subsequently inoculated in white mice and detected in the animals’ blood during post-inoculation day 4 [67]. Meanwhile, *Ornithodoros* ticks, locally known as “chirivicos,” “berrinches,” “cuescas” or “chinches,” were suspected as probable vectors because they were found inside miners’ dwellings, hiding in the walls and floor cracks [67, 70]. Importantly, after the outbreak was controlled, Franco coined the term “spirochetal fever” for the STBRF cases in Muzo Municipality because many patients presented only one febrile episode without relapses [67, 70].

Although *O. turicata*, a tick with distribution in the USA and Mexico, was considered at that time to be the probable vector of the disease in Muzo Municipality, the identity of the vector of the Colombian STBRF remained controversial. In 1921, Emile Brumpt, a physician in the Paris Academy of Medicine, received *Ornithodoros* specimens sent by Franco and described a novel species, *Ornithodoros venezuelensis* [71]. Emile Brumpt also isolated a spirochete from those ticks and named the etiological agent of Colombian and Venezuelan STBRF as *Borrelia venezuelensis*. Yet, a synonymy with *O. rudis* was noted years later, so the name *O. venezuelensis* is now obsolete [55].

In 1927 the North American entomologist Lawrence H. Dunn called attention to the misidentifications of *O. turicata* and *O. talaje* in Panama, Colombia and Venezuela, and stated that the vector of STBRF in those countries corresponded to *O. rudis* [72]. Dunn also pointed out that despite *O. talaje* being found in human dwellings and carrying RFGB, the main host were rats and the tick rarely bites humans, unlike *O. rudis* that avidly parasitizes humans when preent inside dwellings [72]. Therefore, the reports of *O. talaje* from Colombia should be considered as doubtful and rather attributed to *O. puertoricensis*, a species morphologically similar that does occur inside human habitations [73].

Dunn was interested in STBRF in Colombia and joined a yellow fever campaign to study the disease in several departments of the country. He was aware of the suspected cases of STBRF diagnosed by Henry Hanson in patients from Bucaramanga Municipality (Santander Department) [74, 75]. Between July 1923 and July 1924, Dunn visited different departments (Antioquia, Santander, Nariño, Valle del Cauca, Tolima, Cundinamarca, Atlántico, Chocó and Boyacá) and collected a total of 4880 specimens of *O. rudis*. Of these, 61 pools (2483 ticks) were further evaluated for the presence of *Borrelia* using the murine model, with 17 of them ultimately testing positive (28%) [74, 75]. During the expedition, Dunn focused his attention on rudimentary houses with cracks in the walls as a potential shelter for *O. rudis*. Tick collection concentrated in the Colombian Pacific region where malaria is endemic, suggesting that STBRF could be easily confused with malaria [74, 75]. Colloquial names for *O. rudis* varied in accordance with the region in Colombia as follows: “cuescas” in Bucaramanga Municipality, “chinche de la tierra” in Girardot Municipality, “turicata” in Honda Municipality, “berrinche” along the Magdalena River regions, and “chirivico,” “chinche garrapata,” “chinche criolla,” “petacón” and “chinche sin olor” across the Pacific departments [74].

A detailed description of STBRF cases in the Department of Chocó (Colombian Pacific Region) was provided in late 1920s by the physician Emilio J. Pampana [76]. Pampana described a total of 91 cases between 1923 and 1927 diagnosed through microscopic visualization of spirochetes in blood smears. Twenty-nine patients were foreigners (American [US] or European) and 62 were Colombian; 85% of cases occurred in males and 15% in females [76]. Of the 91 patients, only 38 were followed throughout the onset of symptoms until 1 month after the last febrile paroxysm (Table 1). The duration of febrile periods was on average 64 (range: 36–96) h in foreign patients and 54 (range: 24–96) h in native ones; the first interval between paroxysms lasted an average of 10 (range: 4–27) days [76]. In general, few spirochetes were observed in blood smears, but they were quickly noticeable in blood smears from 16 patients, mainly in children aged < 4 years [76]. Leukocytosis was typically observed, with a maximum of 12,800 cells during the onset of fever, and leukopenia during fever remission. This pattern of leukocytosis was often accompanied by neutrophilia. Leukopenia coincided with increased levels of mononuclear cells and lymphocytes [76]. Although, symptoms classified as unusual were described, such as seizures, rash and abdominal pain suggestive of appendicitis, there were no related deaths [76, 77]. Pampana highlighted the use of the drug neosalvarsan as an effective treatment to prevent new febrile paroxysms and shortening their duration [76].

In 1934, the Colombian physician Manuel Roca García published his medical thesis entitled “Contribución al estudio de la fiebre espiroquetal en Colombia” (Contribution to the study of spirochetal fever in Colombia), which provided relevant information on Colombian STBRF, its etiological agent and the related vector *O. rudis* [68]. Roca García based his observations on his clinical and experimental experience during his career in the municipalities of Villeta and Albán, Cundinamarca Department [68]. He referred to STBRF as an endemic sporadic disease occurring in regions with an average temperature of 22–27 °C and altitude of 800–1600 m a.s.l., where *O. rudis* commonly infested bahareque houses, feeding on inhabitants during the night [68]. He described the life-cycle of *O. rudis* under laboratory conditions, observing that it took 3–4 months for larvae to develop into adults, with three nymphal instars, and that larvae and the first nymphal instar needed a meal to achieve molting [68]. While adult ticks spent 1 h for a complete blood meal, larvae and nymphs needed less time. In humans, a papular ecchymotic lesion developed after the tick bite [68].

Roca García described the etiological agent (probably *B. venezuelensis*) as a spirochete with a length of 4–22 (average: 11) µm provided by 2–14 (average: 6) spirals, which was barely visible in thin blood smears; he subsequently improved its visualization using thick blood smears [68]. After experimental subcutaneous or intraperitoneal inoculation of positive human blood into test animals, he noted that dogs, adult guinea pigs and rabbits were not susceptible (absence of spirochetemia), while spirochetes could be visualized in the blood smears of young guinea pigs and rabbits, wild rats and white rats (Table 2) [68]. Interestingly, white rats inoculated with human blood collected during afebrile phases served to predict a new febrile paroxysm in humans; thus, he proposed that white rats be used as a sensitive model to anticipate new relapses in humans and to diagnose the end of the disease when no spirochetes were observed after inoculation [68].

**Table 2:** Dynamics of infection in animals experimentally inoculated with spirochetemic blood collected from humans with soft tick-borne relapsing fever in Colombia [68]

Animal (*n*)^a^	Collection of the inoculated blood sample (febrile/afebrile period)	Days to first observation of spirochetes in blood^b^	Consecutive days of visible spirochetes in blood^b^	Relapsing occurrence (no. of animals with relapsing)
Wild rat, undetermined species (2)	Febrile	2	3	No relapse
Young guinea pig (3)	Febrile	2–4	1	No relapse
Young rabbit (1)	Febrile	1	1	No relapse
White rat (6)	Febrile	1	3	Yes (2)^d^
White rat (2)	Afebrile^c^	4	1–4	Yes (2)^e^

^a^All animals were inoculated subcutaneously or intraperitoneally with samples of spirochetes-positive human blood (0.5–1.0 mL)

^b^By thick blood smear

^c^Between first and second febrile episode

^d^At 4 days after the last spirochete detection

^e^At 2–5 days after the last spirochete detection

Based on the observation of 22 patients (15 naturally and 7 experimentally infected) between July 1932 and May 1934, Roca García described STBRF as a febrile disease with an incubation period of 6–8 days and two clinical forms: “spirochetal fever with relapsing paroxysms” and “spirochetal fever without relapsing paroxysms” [68]. The former was more frequent in “no native” patients, characterized by an abrupt onset of fever, malaise, headache, myalgia, arthralgia, conjunctival hyperemia, vomiting, hepatosplenomegaly and mild jaundice. This first febrile episode lasted for an average of 2–4 days, terminating with a crisis associated with shaking chills, followed by an asymptomatic period (4–8 days) and by two to five relapses. The second paroxysm usually lasted 2 days and was associated with mild symptoms, and was cured in most of the patients. In exceptional cases, patients presented three to five febrile relapses [68]. In comparison, the “spirochaetal fever without relapsing paroxysms,” also called the “benign form,” had a unique febrile period with mild symptoms and was more frequent in native patients or in individuals living for several years in a given endemic region [68]. Overall, of the 22 infected patients, nine (41%) presented one febrile paroxysm, nine (41%) presented two febrile paroxysms, two (9%) presented three febrile paroxysms and two (4.5%) presented four and five febrile paroxysms, respectively (Table 1) [68]. Finally, Roca García described Colombian STBRF as a non-severe disease without fatal consequences, and with anterior uveitis as the most frequent complication. Moreover, despite the disease being susceptible to treatment with arsenic-derived drugs, it also had a favorable course without therapy [68].

Another interesting medical thesis on Colombian STBRF was published by Ángel María Romero García in 1940, which, to our knowledge, constitutes the last description of human cases of STBRF in Colombia [78]. Romero García described six patients (4 men and 2 women), most of whom were from the Departments of Caldas and Tolima, who showed symptoms of acute febrile illness associated with splenomegaly and history of *Ornithodoros* bites; one patient presented *Plasmodium vivax* co-infection [78]. The diagnosis was easily made through direct microscopic visualization of borreliae using Burri's staining (Chinese or Indian ink staining), and all patients were treated with arsenic-derived drugs [78].

Regarding RFGB associated with wild animals, Marinkelle and Grose found large amounts of spirochetes in the blood of a bat (*Natalus tumidirostris*) inside the Macaregua cave (Curití Municipality, Santander Department) and suggested that the agent belonged to RFGB [79]. Further data on this microorganism was not published until recently. We screened blood from 46 bats captured in the Macaregua cave using a genus-specific real-time PCR and detected the *Borrelia 16S* rRNA gene [80]. Positive samples were submitted to a battery of PCRs with the aim to amplify the *Borrelia 16S* rRNA, *flaB, glpQ, p66, ospC, clpA, clpX, nifS, pepX, pyrG, recG, rplB* and *uvrA* genes, but only *flaB* amplicons were obtained [80]. Nucleotide and amino acid sequences of four *flaB* haplotypes were found to be phylogenetically closer to the *B. burgdorferi* s.l. group than to the RFGB [80]. Although isolation and thorough genetic analyses are still pending, our results suggest that the *Borrelia* genotypes characterized from bats roosting in the Macaregua cave might constitute a novel group within the genus.

Considering the work by the medical entomologist Ernesto Osorno Mesa in 1940 [81], as well as previously published papers, the geographical distribution of *Ornithodoros* species acting as probable vectors of RFGB in Colombia is as follows (Fig. 1; Additional file 1: Table S1): *Ornithodoros rudis* in the departments of Boyacá, Santander, Nariño, Valle del Cauca, Atlántico, Chocó, Antioquia, Tolima, Cundinamarca, Risaralda, Cauca, Caldas, Quindío, Caquetá, Huila, Meta and Norte de Santander [55, 67, 68, 70, 74, 76–78, 81, 82]; *O. puertoricensis* in the departments of Atlántico, Córdoba, Sucre and Antioquia [72, 73, 83 – 86); and *Ornithodoros furcosus* in the department of Nariño [87].

### Relapsing fever in Venezuela

The first documented case of STBRF in Venezuela was reported in Caracas by the physicians Taylor and R. Pino Pou in 1918 [88–91]. The patient was from San Cristobal Municipality (Táchira State) and had a history of “night-bug” bites, with symptoms of a febrile illness associated withto chills, arthralgia, myalgia, hepatosplenomegaly and conjunctival hyperemia [90]. Although malaria was the first preliminary clinical diagnosis, visualization of spirochetes in the blood smear confirmed STBRF [90]. Overall, after two relapses the patient fully recovered [90]. More autochthonous cases were described in the states of Táchira, Bolivar and Trujillo by Drs. Bello, Sánchez, Toledo Rojas, Fernández, Murillo and Tejera. The latter physician also demonstrated the role of *Ornithodoros* ticks (probably *O. rudis*), called as “Cuescas” by the native population, as vectors of the Venezuelan *Borrelia* [90–94].

In 1921, Pino Pou published an extensive document describing many aspects of Venezuelan STBRF [90]. He referred to the disease as a mild febrile illness in which spirochetes were barely present upon direct microscopic visualization with Romanowsky stain, but which were easily observed using Burri's stain [90]. Inoculation of spirochetemic human blood into laboratory animals successfully infected rats and mice, but monkeys, dogs, chickens, guinea pigs and rabbits were resistant to the Venezuelan *Borrelia* [90]. Pino Pou also described 21 human cases (including the first observed in 1918), with most of them from Táchira State, with symptoms such as fever, chills, headache, arthralgia and myalgia [90]. Six patients presented one febrile paroxysm, two patients presented one febrile relapse, seven patients presented two febrile relapses and four patients presented three febrile relapses (Table 1) [90]. Interestingly, two patients presented anterior uveitis and two others presented malaria co-infection [90].

Regarding animal hosts which could participate in the ecoepidemiology of STBRF in Venezuela, Pifano studied synanthropic rodents (*R. norvegicus*, *Mus musculus*) and opossums in the search for spirochetes in thick blood smears. Remarkably, only opossums were positive [91, 95], indicating these animals as potential reservoirs of spirochetes. However, further investigations have not been performed in this country.

Considering previous published papers, the geographical distribution of *O. rudis* in Venezuela is in Táchira State (Fig. 1; Additional file 1: Table S1) [90, 92, 93].

### Relapsing fever in Peru

The evidence regarding relapsing fever spirochetes in Peru is limited to LBRF. “Relapsing typhus,” the local name for LBRF in Peru, was clinically described for the first time by the physician Demetrio García del Barco in February of 1917 while he was investigating an outbreak of undifferentiated febrile illness at Tambo (Ayacucho Department) [96]. The disease was microbiologically confirmed by the physicians Ramon Ribeyro, Abel Olaechea and Julio Gastiaburu, who observed spirochetes in the blood of febrile patients [96]. In the same year, the disease was also confirmed in other regions of Ayacucho Department: in Junín and Arequipa Departments, together with epidemic typhus (*R. prowazekii* infection), by Dr. Miguel Escarcena; in Cuzco Department, by Dr. Augusto Belaunde; and in 1918 in Huancavelica Department, by Dr. Leoncio Pajuelo [96]. A paper published in 1920 by Eliodoro Del Prado stated that LBRF in Peru was a widely distributed disease and endemic mainly in the indigenous population of the Andes, with sporadic dissemination to coastal and Amazonian regions [96]. The departments of Ayacucho, Huancavelica and Junín were considered hotspots for LBRF based on clinical and microbiological descriptions. The departments of Cajamarca, Ancash, Lima, Arequipa, Cuzco, Apurimac and Puno were regarded as secondary hotspots, based only on clinical features [96]. As in other parts of the world, the human clothing lice *P. humanus humanus* was confirmed as the vector based on: (i) the observation of spirochetes after crushing the ectoparasites present in the patients’ clothes; (ii) the habit of the indigenous population to crush lice with nails; and (iii) the absence of ticks inside human dwellings [96].

Del Prado clinically described Peruvian LBRF as a febrile disease with an incubation period of 2–10 days and two febrile paroxysms [96]. The first febrile paroxysm typically began as an abrupt onset of fever with nonspecific symptoms, such as chills, intense headache, dizziness, arthralgia, myalgia, nausea, conjunctival hyperemia, vomiting, hepatosplenomegaly, mild jaundice and, in some cases, petechial rash involving the trunk [96]. This first febrile episode lasted for an average of 5–6 days, terminating with a crisis associated with shaking chills, followed by an asymptomatic period (6–8 days) and then by the second and last relapse of similar or even milder symptoms [96]. Severe manifestations were also described, including gastrointestinal hemorrhage, epistaxis, coma, seizures and, in pregnant women, abortion or stillbirth [96]. The mortality rates among patients whose disease was untreated ranged from 4% to 6%; however, when treatment with arsenic-derived drugs was administered, the death rate was null [96]. As in STBRF, diagnosis was made through direct microscopic visualization of borreliae during the febrile period [96].

Although STBRF has not been identified in Peru, soft ticks do bite humans and infest dwellings. Indeed, in 1957 Herrer and Morales investigated soft ticks in rural areas in different Peruvian departments (Cajamarca, Amazonas, Piura) [97]. Overall, 1655 *O. furcosus* specimens (nymph and adults) were fed or inoculated (macerated ticks) into 80 white mice and 25 guinea pigs [97]. Thick blood smears of the animals stained with Giemsa were examined daily for the presence of spirochetes during 24–28 days, yet all of the animals were negative [97]. Thus, the role of *O. furcosus* as a RFGB vector is still obscure and needs further investigation. Noteworthy, given their morphological similarity, identifications of *O. turicata* in Colombia and Venezuela could actually represent *O. furcosus*.

In March 1998, a serological survey in which micro-immunofluorescence tests were used to detect immunoglobulin G (IgG) antibodies against *R. prowazekii*, *Bartonella quintana* and *B. recurrentis* was performed in 194 individuals from rural areas of Calca Province (Cuzco Department) [98]. The results showed 20, 12 and 1% reactiveness, respectively [98]. Additionally, human clothing lice were collected on 16 individuals, and only *B. quintana* DNA was detected after species-specific PCR analyses [98]. The presence of antibodies to any of these three louse-transmitted microorganisms was significantly associated with louse infestation [98].

Considering previous published papers, the presumed geographical distribution of *P. humanus humanus* related to LBRF cases in Peru is as follows (Fig. 1; Additional file 1: Table S1): Cuzco, Ayacucho, Arequipa, Huánuco, Huancavelica, Pasco, Junín, Cajamarca, Ancash, Lima, Apurímac and Puno departments [96, 98]. The presumed geographical distribution of *O. furcosus* in Peru is depicted in the map as the departments of Cajamarca, Amazonas and Piura (Fig. 1; Additional file 1: Table S1) [97].

### Relapsing fever in Brazil

The first study on RFGB in Brazil was performed in 1951 by the Gordon E. Davis, who had received from Henrique Aragão 31 specimens (6 females, 3 males and 22 nymphs) of *Ornithodoros brasiliensis* (locally known as “dog tick” or “Mouro bug”) collected in São Francisco de Paula Municipality (Rio Grande do Sul State) in the soil around houses, domestic animal shelters and dens of skunks (*Conepatus* sp.) [99]. In his laboratory, Davis allowed the ticks to feed individually upon white mice in order to recover spirochetes. A nymph fed upon one mouse was infected since spirochetes were observed in blood of the positive animal on days 6, 10 and 11 days after feeding [99]. Blood from the spirochetemic animal was inoculated into two white mice and two guinea pigs. After the seventh passage on mice with relapsing episodes, the spirochete was lost and not recovered again from the original tick. In the meantime, spirochetes did appear in the peripheral blood of guinea pigs between the fifth and seventh days, accompanied with fever [99]. Davis proposed the name *Borrelia brasiliensis* n. sp. [99], yet despite current efforts, the spirochete has never been detected or observed again.

Interestingly, Davis stated that although STBRF had not been reported in Rio Grande do Sul State; however, in 1931 Pinto and Primio reported headache, dyspnea and high body temperature in humans bitten by *O. brasiliensis* [99]. Nowadays, we know that *O. brasiliensis* causes human and animal toxicosis [100–103] and that clinical manifestations resemble those described by Pinto and Primio [99, 102].

After more than 60 years without any new developments, in 2017 our group collected 30 *Ornithodoros* specimens (15 females, 10 males and 5 nymphs) between debris of bird nests inside hollow palm-trees in Riachão Municipality (Maranhão State) [104]. The ticks were morphologically identified as *O. rudis*. With this finding, seminal evidence on *O. rudis*’s natural history was unveiled, since previous reports mentioned this tick only in association with poultry and human dwellings [104]. Attempts to isolate spirochetes from the ticks were successful using Vesper mice (*Calomys callosus*); only one female tick was positive for borrelial infection, and spirochetes were recovered from mice blood on the fourth day [104]. Isolation in BSK medium was performed, and we subsequently characterized the* 16S* rRNA, *flaB* and *glpQ* genes. A phylogenetic analysis confirmed that *B. venezuelensis* harbored by *O. rudis* from Maranhão State is closely related to *B. turicatae* [104]. *Borrelia venezuelensis* RMA01 constitutes to date the sole isolate of a RFGB transmitted by an *Ornithodoros* tick in South America.

Recently, we conducted collections of human-biting *Ornithodoros* species in natural ecosystems and inside human dwellings in six Brazilian states (Ceará, Goiás, Mato Grosso, Mato Grosso do Sul, Maranhão and Rondônia) [105]. Eight species were collected (*O. rudis*, *Ornithodoros mimon*, *Ornithodoros hasei*, *Ornithodoros rietcorreai*, *Ornithodoros tabajara*, *Ornithodoros rostratus*, *Ornithodoros marinkellei* and *Ornithodoros fonsecai*), of which four were positive for *Borrelia* DNA [105]. With high support values, Bayesian phylogenetic analyses showed that the *Borrelia* spp. characterized from *O. mimon*, *O. rietcorreai* and *O. tabajara* form a monophyletic clade related to RFGB occurring in the Old World, while the *Borrelia* sp. harbored by *O. hasei* clustered within the New World RFGB [105]. Given that these four *Ornithodoros* species harboring putatively new RFGB species do parasitize humans in Brazil [105–108], elucidating STBRF as a possible cause of undifferentiated febrile syndrome is now imperative in the country [109].

Considering previous published papers, the presumed geographical distribution of *Ornithodoros* species that are probable vectors of RFGB in Brazil is as follows (Fig. 1; Additional file 1: Table S1): *Ornithodoros brasiliensis* in Rio Grande do Sul State [99–103, 110]; *O. rudis* in the states of Maranhão and Goiás [104, 105, 111]; *O. mimon* in the states of Minas Gerais, Maranhão, Mato Grosso, Rio Grande do Norte, Pernambuco, Goiás, Ceará and São Paulo [105, 106, 111–113]; *O. hasei* in the states of Ceará, Maranhão, Espírito Santo, Mato Grosso, Mato Grosso do Sul and Amapá [105, 114–116]; *O. rietcorreai* in the states of Ceará, Maranhão, Piauí, Tocantins, Bahia and Paraíba [105, 107, 108, 111, 113, 117–119]; and *O. tabajara* in Ceará State [105, 120].

### Relapsing fever in Bolivia

Limited information is available on STBRF in Bolivia. In 1994, Ciceroni et al. published a seroepidemiological study carried out in three autochthonous Guaraní and mestizos communities (Camiri, Boyuibe and Gutierrez) in Cordillera Province (Santa Cruz Department, south-eastern Bolivia) and determined exposure to *Borrelia* spp. using *B. burgdorferi*, *B. parkeri* and *B. turicatae* antigens and indirect immunofluorescence assays (IFA) [121]. Overall, for a total of 305 sampled individuals, antibodies anti-*B. burgdorferi*, anti-*B. parkeri* and anti-*B. turicatae* were detected in 10.8, 8.2 and 16.1% of individuals, respectively [121]. Because cross-reaction between *Borrelia* spp. was high, IFA-positive serum samples were absorbed with *Treponema phagedenis* and re-tested for anti-*Borrelia* antibodies; residual species-specific antibodies to *B. burgdorferi*, *B. parkeri* and *B. turicatae* were observed in 1% of all sera, respectively [121]. The above findings indicate exposure to RFGB (or eventually to Lyme group borreliae) in the studied Bolivian communities. Nevertheless, since Bolivia is not an endemic area for Lyme borreliosis [122], seropositivity for *B. burgdorferi* should be interpreted with discretion.

In 2009, Parola et al. used carbon dioxide traps to capture triatomines and collected 35 *Ornithodoros* ticks in rocky outcrops located in the Eastern Cordillera, Cochabamba Department, near the town of Cotapachi [123] (Fig. 1; Additional file 1: Table S1). Two ticks were positive for *Borrelia* DNA, and phylogenetic analyses showed that the sequences clustered with those of Old World RFGB [123]. Moreover, phylogenetic analyses from a recent paper show that ticks reported by Parola et al. cluster into a monophyletic group with *Ornithodoros quilinensis*, *Ornithodoros xerophylus* and *Ornithodoros octodontus* [124]. Remarkably, the genetic distance that separates the *Ornithodoros* sp. from Bolivia with *O. xerophylus* is low, a finding which suggests conspecificity.

### Relapsing fever in Chile

To date, human cases of STBRF or LBRF have not been documented in Chile. Nevertheless, two recent studies unveiled evidence on natural foci of RFGB. In 2018, we collected ticks on small mammals and birds within the Río Los Cipreses National Reserve, located in the higher basin of the Cachapoal River, O´Higgins Region (central Chile) [125]. Eight larvae of an *Ornithodoros* sp. morphologically and genetically *affinis* to *Ornithodoros atacamensis* were collected on *Phyllotis darwini* [125, 126]. Four of these larvae were screened and found to be positive for the *Borrelia 16S* rRNA and *flaB* genes [125]. The detected *Borrelia* formed a monophyletic group with “*Candidatus* Borrelia johnsonii,” a recently described pathogenic agent [127], and clustered as a sister group with a RFGB clade composed of *B. parkeri*, *B. turicatae* and *B. venezuelensis* [125]. Moreover, Thomas et al. published a study which surveyed 53 small mammals in four localities belonging to hyper-arid regions from northern Chile (Socoroma, Chusmiza, Pampa del Tamarugal National Reserve and Bosque Fray Jorge National Park), during July 2018 [128]. Sequences of a novel RFGB genotype were recovered from blood samples of two *Phyllotis xanthopygus* rodents collected at Socoroma [128]. Phylogenetic analyses positioned the detected borreliae into a clade with the *Borrelia* sp. characterized from the "*Ornithodoros* sp. Bolivia” [123]. The above results represent the first detection of *Borrelia* spp. DNA in rodents from South America. Evidence for other two STBRF *Borrelia* genotypes circulating in Chilean ecosystems indicates that the seabird soft tick *Ornithodoros spheniscus* and rodent-associated *O. octodontus* could participate in enzootic cycles of RFGB as well [129, 130]. The current distribution of soft ticks potentially associated with RFGB in Chile is depicted in Fig. 1 and includes the de Arica and Parinacota, Atacama, Bernardo O’Higgins and Coquimbo regions (Additional file 1: Table S1).

### Relapsing fever in Argentina

In Argentina, information on RFGB is scarce. To our knowledge, only two papers, both published during the first half of the twentieth century, have described imported and autochthonous cases of LBRF.

In 1911, Pedro J. García, working as a physician at Hospital Mixto de Tucumán (Tucumán Province), described two probable imported cases of LBRF, both diagnosed through blood smears [131]. One patient was a 28-year-old Bulgarian man and the other was a 20-year-old Spanish man, both immigrants to Argentina [131]. The Spanish patient presented two febrile episodes and mild splenomegaly [131].

Years later, Dr. Vicente E. Bernasconi described a probable autochthonous case of LBRF in a Bolivian nurse who worked at Hospital San Roque in San Salvador de Jujuy (Jujuy Province) [132]. This patient developed an abrupt onset of fever accompanied by nausea, chills and splenomegaly. The first febrile episode lasted 3 days, then faded, and a subsequent relapse appeared 1 day later [132]. Finally, after 7 days without symptoms, the patient had a final fever recurrence, when spirochetes were observed in blood smears. Successful treatment with arsenic-derived drugs was achieved [132]. Interestingly, Bernasconi refers to three LBRF historical cases described by Drs. Emilio Lorenz and Paterson [132]. The case of Emilio Lorenz was a Greek patient who apparently contracted the infection in Pehaujó City (Buenos Aires Province), and Dr. Paterson’s cases were two immigrants from Russia who entered the country with the infection [132].

Considering the previous published paper [132], the autochthonous LBRF case in Argentina occurred in the Jujuy Province (Fig. 1; Additional file 1: Table S1).

### Other reports

In addition to the information presented in the preceding sections of this article, we consider the following descriptions also to be relevant since they support the occurrence of RFGB in other Latin American countries, although some have yet to be further confirmed.

1. *Ornithodoros talaje* (published as “*Argas talaje*”) was originally described by Guérin-Méneville in 1849, on specimens collected at “Casa Guastatoya” (appearing as “Casa Vieja de Gastoya” in the publication), in El Progreso Department, Guatemala [133]. Guérin-Méneville described *O. talaje* as a very anthropophilic tick that infested bamboo houses, hiding during the day in wall cracks and biting people at night [133]. Interestingly, as demonstrated with *O. talaje* from Mexico, Gordon Davis described vector competence of Guatemalan *O. talaje* for *B. mazzottii,* and that white mice, white rats, new-born rabbits and hamsters were susceptible to infection, while adult and new-born guinea pigs and young rabbits were refractory [46].
2. *Ornithodoros puertoricensis* was described by Fox in 1947, from specimens collected on rats in San Juan (Puerto Rico) [134]. Interestingly, Fox compared *O. puertoricensis* with allotments labeled as *O. talaje*, and concluded that the alleged “*O. talaje”* collected on rats during the work of Dunn and others in Panama and Colombia might indeed correspond to *O. puertoricensis* [134].
3. *Ornithodoros rudis* and *O. furcosus* are listed as species occurring in Ecuador [46, 87, 97, 135]. However, any implication of these populations of soft ticks with RFGB is currently unknown.
4. A 26-year-old Dutch woman with a 2-day history of fever peaking at 39 °C, cold shivers, generalized myalgia and nausea, which developed after a 2-week trip to Guatemala and Belize, was reported [136]. While the case was being studied, spirochetes were detected in thick blood smears and STBRF was diagnosed. The patient was treated with doxycycline for 7 days and discharged home in good condition [136]. No genetic characterization or isolation of the spirochetes was provided at that time.
5. According to the Pan American Sanitary Bureau, between 1946 and 1968 Latin American countries that reported cases of STBRF were: Argentina (*n* = 32 cases), Bolivia (*n* = 3), Colombia (*n* = 19,296), Ecuador (*n* = 4), Mexico (*n* = 34), Nicaragua (*n* = 10), Panama (*n* = 104) and Venezuela (1069) [137–147]. During this same period, cases of LBRF were reported in Bolivia (*n* = 439), Colombia (*n* = 4), Mexico (*n* = 40) and Peru (*n* = 135) [137–147].

### *Borrelia anserin*a and *Borrelia theileri* in Latin America

The first published record of *B*. *anserina* (named as “*Spirochaeta gallinarum*”) in Latin America is from Brazil, and identification was performed by Marchoux and Salimbeni in 1903 [148]. Subsequently, between 1908 and 1909, Dr. S. Von Prowazek studied the infection dynamics of *B. anserina* in *A. miniatus* and confirmed the role of this soft tick as a biological vector [149]. Also, in Brazil, Henrique Aragão performed methodological studies seeking prophylactic strategies (i.e. serum therapy, vaccines) to prevent what he described as a “devastating epizootic disease” of hen flocks worldwide. High infestation of poultry with *Argas* ticks vanished drastically with the implementation of better breeding techniques and strict arthropod control strategies. Studies on *B*. *anserina* or avian borreliosis were scarce after the 1920s, and very few isolates are currently available worldwide.

At the end of the twentieth century, Labruna et al. successfully cryopreserved a *B. anserina* strain (“PL”) recovered from infected chickens [150]. *Borrelia anserina* strain PL was isolated and cultured in BSK medium and corresponds to the sole isolate for the species currently available in Latin America [151]. An additional study in Brazil experimentally transmitted *B*. *anserina* to domestic chickens using infected *A. miniatus*. The authors recorded the prepatent (5–7 days) and patent periods (4–7 days) and described the absence of borreliae in blood smears between the 13th and 25th day of infection [29]. Infected animals presented ruffled feathers, pale combs, drowsiness, greenish diarrhea and inappetence during the spirochetemic period [29]. Another experimental study in Brazil assessed hematological abnormalities in animals exposed to infected ticks, which developed normocytic normochromic anemia, leukocytosis with heterophilia and monocytosis concomitant with the spirochetemia [152]. A related work registered hepatic alterations in experimentally infected fowls and found increased levels of hepatic enzymes (i.e. ALT, AST), gross pathological lesions (i.e. moderate hepatomegaly, congestion, irregular surface, red to cyanotic appearance) and different histopathological abnormalities (i.e. mononuclear inflammatory infiltrates, fibrinoid necrotic foci, dilatation of sinusoids and vacuolation of hepatocytes) [153].

Despite strict prophylaxis and control measures in laying hens and commercial breeding broiler flocks, it is worthy to note that the advent of free-range husbandry systems with better welfare conditions would potentially allow the establishment of *Argas* populations and favor the re-emergence of fowl spirochetosis [151].

*Borrelia theileri* was firstly described by Nájera et al. in 1949 [154], then by Ibáñez and Laffont in 1959 [155] and further by Hadani et al. in 1985 [156], in cattle from the northern region of Argentina. In 1987, Guglielmone et al. described a spirochete compatible with *B*. *theileri* infecting *R.* (*B.*) *microplus* ticks in Tucumán Province [157]. Recently, the first morphological and molecular characterization of this spirochete was achieved in heifers from Chaco Province through microscopic examination of thick blood films and amplification of the *flaB* gene [34].

In 1978, in Mexico, Smith et al. registered *B*. *theileri* while studying *Babesia bovis* in *R.* (*B.*) *microplus* ticks. These authors found this spirochete in field-collected ticks and described its presence within adult ticks and eggs, suggesting a transovarial transmission route [158].

In 1996, in Brazil, Martins et al. published the first description of *B*. *theileri* in *R.* (*B.*) *microplus* ticks from Rio Grande do Sul [159]. These authors observed the spirochete in the hemolymph of one female tick (with 10 days of engorgement) without pathogenic effects or fitness reduction. A subsequent study performed the first molecular identification of this *Borrelia* in DNA extracted from a single *R.* (*B.*) *microplus* tick collected from a horse in Minas Gerais State (southeastern region) [160]. Recently, Cordeiro et al. performed a morphological, molecular and phylogenetic characterization of *B. theileri* in engorged females from a *R.* (*B.*) *microplus* colony (Porto Alegre strain) [161]. These authors found a 2% infection rate (1/50), based on hemolymph smears and amplification of borrelial genes (*glpQ, hpt* and *flaB*), which confirmed the species, which was named *B*. *theileri* strain C5 [161].

*Borrelia* *theileri* is likely to be widely distributed throughout Latin American countries, with the bacteria perpetuating in transmission cycles that involve cattle (and probably other ruminants and horses) and *R.* (*B.*) *microplus* ticks. Because cattle infection is commonly asymptomatic or presents unspecific clinical signs (i.e. fever, lethargy and anemia) [4], it is particularly challenging to identify the disease in natural conditions.

## Conclusions and future perspectives

Relapsing fever group *Borrelia* in Latin America have not disappeared; rather, they constitute an emerging group of bacteria that should received more attention. Although the identity of the vector is clear for some borreliae, taxonomic and genetic studies are needed to clarify which species of soft ticks transmit RFGB to humans in Colombia, México and Panama. In these three countries, as well as Venezuela, STBRF was once studied; however, interest in studying the disease is currently faint and, therefore, updated information on its epidemiology unavailable. Recently, the finding and isolation of *B. venezuelensis* in northeastern Brazil has ignited medical and scientific interest in the disease. The study of STBRF in Latin America must now focus in obtaining isolates of *B. venezuelensis* in countries where the agent was once endemic, as well as isolating the recently identified *Borrelia* spp. associated with human-biting *Ornithodoros*. Such isolates are needed not only to obtain sound genetic information but also to design serological assays using local strains.

Wild animals involved as a reservoir of STBRF group borreliae have been barely studied, and data on the ecoepidemiology of different strains for which genetic information is available still need considerable research. *Borrelia anserina* and *B. theileri* are transmitted by species of ticks with a vast distribution in the continent, and the few records available on these agents underestimate their real geographical range.

Regarding LBRF, countries that include the Andes range within their territories seem to have had concentrated cases of *B. recurrentis* in the past. The most vulnerable settlements in Latin America are located along the cold side of the Andes mountains. Although the disease has not been reported in peer-reviewed papers or medical documents since the first half of the twentieth century, its occurrence should not be dismissed, but rather investigated in autochthonous populations living in mountainous environments. Finally, acute febrile illnesses are common in tropical and subtropical regions of the continent, so STBRF and LBRF should be considered to be differential diagnoses.

## Supplementary Information

**Additional file 1: Table S1**. Data on human cases and/or probable arthropod vectors related to relapsing fever group borreliae in Latin America.

## Data Availability

Geographical coordinates to construct the maps of Fig. 1 are available in the Additional file 1: Table S1.
